# Eine klinische Perspektive auf die Behandlung von chronischer myeloischer Leukämie in der chronischen Phase

**DOI:** 10.1159/000529704

**Published:** 2023-02-22

**Authors:** Valentin García-Gutiérrez, Massimo Breccia, Elias Jabbour, Michael Mauro, Jorge E. Cortes

**Affiliations:** ^a^Servicio Hematología y Hemoterapia, Hospital Universitario Ramón y Cajal, Instituto Ramón y Cajal de Investigación Sanitaria (IRYCIS), Madrid, Spanien; ^b^Department of Translational and Precision Medicine, Sapienza University, Rom, Italien; ^c^The University of Texas MD Anderson Cancer Center, Houston, Texas, USA; ^d^Memorial Sloan Kettering Cancer Center, New York, New York, USA; ^e^Georgia Cancer Center, Augusta University, Augusta, Georgia, USA

**Keywords:** Chronische myeloische Leukämie, Tyrosinkinase-Inhibitoren, Erstlinienbehandlung, Behandlungswechsel, behandlungsfreie Remission

## Abstract

Tyrosinkinase-Inhibitoren (TKIs) haben die langfristigen Behandlungsergebnisse für Patienten mit chronischer myeloischer Leukämie (CML) erheblich verbessert. Nach Imatinib (einem TKI der ersten Generation) wurden TKIs der zweiten und dritten Generation entwickelt. Mit 5 TKIs, die auf *BCR::ABL* abzielen und in den meisten Ländern zugelassen sind (Imatinib, Dasatinib, Bosutinib, Nilotinib und Ponatinib), und der kürzlich erfolgten Zulassung von Asciminib in den USA sind die Behandlungsentscheidungen komplex und erfordern eine Bewertung patientenspezifischer Faktoren. Optimale Behandlungsstrategien für CML entwickeln sich weiter, wobei der Schwerpunkt verstärkt darauf liegt, ein tiefes molekulares Ansprechen zu erzielen. Anhand von klinisch relevanten Fallstudien, die von den Autoren dieses Reviews entwickelt wurden, diskutieren wir 3 Hauptszenarien aus der Perspektive internationaler Experten. Erstens untersucht dieser Übersichtsartikel patientenspezifische Merkmale, die die Entscheidungsfindung zwischen TKIs der ersten und zweiten Generation bei der Erstdiagnose von CML beeinflussen, einschließlich der Begleiterkrankungen des Patienten. Zweitens wird eine gründliche Bewertung der therapeutischen Optionen im Falle eines Versagens der Erstlinienbehandlung (wie in den Richtlinien des National Comprehensive Cancer Network und des European LeukemiaNet definiert) diskutiert, zusammen mit praktischen Erwägungen zur Überwachung des optimalen Ansprechens auf die TKI-Therapie. Drittens verdeutlicht diese Übersichtsarbeit die Erwägungen zum und die Bedeutung des Erreichen(s) einer behandlungsfreien Remission als Behandlungsziel. Aufgrund des Zeitpunkts der Erstellung befasst sich dieser Review auch mit den globalen Herausforderungen, denen Hämatologen bei der Behandlung von CML-Patienten während der COVID-19-Pandemie häufig gegenüberstehen. Da bei CML weiterhin neue Behandlungsansätze erforscht werden, erörtert diese Übersichtsarbeit schließlich auch das Aufkommen neuerer Therapien wie Asciminib. Dieser Artikel kann eine nützliche Referenz für Ärzte sein, die CML-Patienten mit TKI der zweiten Generation behandeln, und kann, da er sich auf die internationalen und persönlichen Erfahrungen der Ärzte konzentriert, einen Einblick in alternative, zuvor nicht in Betracht gezogene Ansätze geben.

## Einführung

Die weltweite Inzidenz der chronischen myeloischen Leukämie (CML) im Jahr 2017 betrug 34 179, mit insgesamt 24 054 CML-bedingten Todesfällen [1]. Das Aufkommen von Tyrosinkinase-Inhibitoren (TKIs) hat die Behandlungslandschaft erheblich verändert und die Therapieergebnisse für Patienten mit CML verbessert: Die Behandlung mit Imatinib, dem TKI der ersten Generation (1G), hat die 8-Jahres-Gesamtüberlebensrate von 20% auf 87% verbessert [2]. 6 TKIs sind zugelassen und werden häufig zur Behandlung von CML eingesetzt: Imatinib, die TKIs der zweiten Generation (2G) Dasatinib, Nilotinib und Bosutinib, der TKI der dritten Generation (3G) Ponatinib sowie der neuartige TKI, der Erste seiner Klasse, der speziell auf die ABL-Myristoyl-Tasche (STAMP-Inhibitor) abzielt: Asciminib [3, 4, 5, 6, 7]. Mit den derzeit verfügbaren und sich entwickelnden TKIs können Patienten mit CML eine durchschnittliche Lebenserwartung haben, die der der Allgemeinbevölkerung nahekommt [8, 9], und dies hat die Gesamtprävalenz trotz der relativ niedrigen Inzidenzrate signifikant erhöht [10]. Obwohl sie aus derselben Wirkstoffklasse stammen, unterscheiden sich alle TKIs in Bezug auf ihre Wirksamkeit, unerwünschte Ereignisse (UEs) und ihre Wirksamkeit gegen *BCR::ABL*-Mutationen. Bei der Entscheidung für die ideale TKI-Therapie müssen Ärzte und Patienten daher viele Faktoren berücksichtigen.

In diesem Review untersuchen wir die Umstände, unter denen Ärzte einen 2G-TKI in Betracht ziehen würden. Anhand von Fallstudien, die von den Autoren dieser Übersichtsarbeit entwickelt wurden, und unter Berücksichtigung klinischer Erfahrungen, Patientencharakteristika und praktischen Überlegungen diskutieren wir Behandlungsentscheidungen zu 2G-TKIs. Dieser Review konzentriert sich auf 5 Themen in der Behandlung von CML: die Erstlinien (1L)-Behandlung, frühe Umstellung und Überlegungen zur Überwachung des optimalen Ansprechens, klinische Überlegungen zur behandlungsfreien Remission (TFR), die Behandlung von CML während der COVID-19-Pandemie und die Auswirkungen neu entstehender TKIs.

## 1L-Behandlung und TKI-Auswahl

Entscheidungen zur Erstbehandlung sind komplex und umfassen krankheits- und patientenspezifische Faktoren, zusätzlich zu anderen Faktoren wie Verfügbarkeit, Dosierungsplan, Kosten und dem Vorhandensein von Komorbiditäten. Obwohl Imatinib nach wie vor der am weitesten verbreitete TKI für die neu diagnostizierte (ND) CML in der chronischen Phase (CML-CP) ist, haben klinische Studien gezeigt, wie die 1L-Anwendung von 2G-TKIs das Ansprechen verbessern und das Fortschreiten der Krankheit verlangsamen kann. Ihr Einsatz muss jedoch gegen potenzielle Risiken und Kosten abgewogen werden.

### Fallstudie 1

Bei einem 65-jährigen männlichen Patienten wurde eine Hochrisiko-CML (European Treatment and Outcome Study Long-Term Survival (ELTS)-Score von 2,53 und Sokal-Score von 1,9) diagnostiziert, nachdem sein Hausarzt bei einer routinemäßigen Nachsorge eine erhöhte Anzahl weißer Blutkörperchen von 58 × 10^9^/l festgestellt hatte. Bei der Diagnose hatte der Patient eine Milzgröße von 5 cm unterhalb des Rippenrandes und 8% Blasten im peripheren Blut. Eine Analyse des Bandenmusters der Chromosomen dokumentierte nur das Philadelphia-Chromosom. Der Patient hat eine überwiegend sitzende Lebensweise, ist übergewichtig und hat eine lange Vorgeschichte von Diabetes mellitus, der mit Metformin nicht gut kontrolliert werden konnte. Er hatte vor 5 Jahren einen Myokardinfarkt, aber ohne weitere Episoden und hat seitdem mit dem Rauchen aufgehört. Der Patient bevorzugt ein kostengünstiges Medikament.

#### Überlegungen der Ärzte zum Behandlungsansatz

Die Behandlungsstrategien für CML konzentrieren sich zunehmend auf das Erreichen eines schnell einsetzenden, anhaltenden, tiefen molekularen Ansprechens (DMR; molekulares Ansprechen mit einer 4,5-log-Reduktion von *BCR::ABL1* (MR^4,5^) auf der internationalen Skala) mit einer 1L-Behandlung. Die Leitlinien des National Comprehensive Cancer Network (NCCN) und die Empfehlungen des European LeukemiaNet (ELN) betrachten das Erreichen eines frühen molekularen Ansprechens (EMR; *BCR::ABL1* < 10% innerhalb von 3 Monaten) als Behandlungsmeilenstein für ein optimales Ansprechen. Daher ist dies eine wichtige Überlegung bei der Auswahl eines 1L-TKI.

#### Auf klinischen Studien basierende Überlegungen: Risikobewertung und Wirksamkeit

Die NCCN-Richtlinien und ELN-Empfehlungen schlagen 1L-2G-TKIs für Patienten mit niedrigen oder möglicherweise mittleren Risikowerten vor, basierend auf ihren besseren Ansprechergebnissen im Vergleich zu Imatinib [11]. Darüber hinaus schlagen die ELN-Empfehlungen vor, anstelle der Hasford/Sokal-Risikoscores das neue ELTS-Scoringsystem zu verwenden, um die CML-Ausgangswerte zu bewerten [11], da es die krankheitsspezifische Mortalität und das molekulare Ansprechen vorhersagen kann [12]. Obwohl Patienten, die in wichtige Studien aufgenommen wurden, zur Entwicklung des ELTS-Scoringsystems herangezogen wurden, wurde der ELTS-Score noch nicht prospektiv in zulassungsrelevanten Studien mit 1L-TKIs verwendet. Die NCCN-Richtlinien empfehlen die Verwendung des Sokal-Scores [13], der auch außerhalb der USA weit verbreitet ist, aber der ELTS-Score ist nützlich, um das Langzeitüberleben zu beurteilen, insbesondere bei jüngeren Patienten. Als Kliniker verwenden wir oft beide Scoringsysteme.

Wichtige klinische Studien wie DASISION (Dasatinib vs. Imatinib), ENESTnd (Nilotinib vs. Imatinib) und BFORE (Bosutinib vs. Imatinib), in denen die meisten Patienten CML mit mittlerem oder hohem Risiko hatten, zeigten überlegene Raten kompletter zytogenetischer Remission (CCyR) und ein gutes molekulares Ansprechen (MMR) mit Dasatinib, Nilotinib und Bosutinib in der Erstlinie gegenüber Imatinib (Tab 1) [14, 15, 16]. Das MMR nach 12 Monaten war bei allen drei 2G-TKIs im Vergleich zu Imatinib signifikant höher und blieb über die Langzeitnachbeobachtung erhalten [17, 18, 19]. Allerdings führte keiner der 2G-TKIs (mit Ausnahme von Nilotinib bei 400 mg) zu einer signifikanten Verbesserung des Gesamtüberlebens oder des progressionsfreien Überlebens im Vergleich zu Imatinib, was bedeutet, dass ein früheres Ansprechen und verbesserte Ansprechraten mit 2G-TKIs nicht unbedingt zu einer Verbesserung des Gesamtüberlebens und des progressionsfreien Überlebens führten. Darüber hinaus war es für etwa 40–60% der mit 2G-TKIs behandelten Patienten unwahrscheinlich, dass sie eine MR^4,5^ erreichten, wobei ein Plateau nach etwa 5 Jahren beobachtet wurde [17, 19, 20].

Da der Patient in dieser Fallstudie eine Hochrisiko-CML hat, wäre ein 2G-TKI am besten für die 1L-Behandlung geeignet. Unabhängig vom Risikoscore sollten auch jüngere Patienten [21] und solche mit seltenen Transkripten [22] als Hochrisikopatienten betrachtet werden, und ein 2G-TKI kann die bevorzugte Option sein. Patienten mit CML mit niedrigem Risiko können ebenfalls von einer Behandlung mit einem 1L-2G-TKI profitieren. Im 5-Jahres-Bericht der BFORE-Studie betrug die MR^4,5^-Rate für Patienten mit niedrigem Sokal-Risikoscore 53,7% mit Bosutinib gegenüber 42,5% mit Imatinib [19]. Daher sollte die Wahl des TKI nicht nur auf Risikobewertung und Wirksamkeit beruhen, sondern sollte auch auf jeden Patienten zugeschnitten sein und gegen die Therapieziele abgewogen werden.

#### Auf klinischen Studien basierende Überlegungen: Komorbiditäten

Die Verwendung von 1L-2G-TKIs muss gegen ihre potenziellen Risiken abgewogen werden. Daher sollten Kliniker auch die bekannten UEs im Zusammenhang mit 2G-TKIs und Komorbiditäten von Patienten zu Studienbeginn berücksichtigen. Komorbiditäten von Patienten werden häufig anhand des Charlson Comorbidity Index (CCI) bewertet, und das Überleben von Patienten mit CML nimmt mit steigendem CCI-Score ab [18], wobei das Sterberisiko hauptsächlich von den Komorbiditäten bestimmt wird [23]. In DASISION war Dasatinib in allen CCI-Untergruppen mit verbesserten Ergebnissen gegenüber Imatinib verbunden, wobei ein signifikanter Unterschied in den MR^4,5^-Raten verbunden mit einem höheren Komorbiditätsscore auf eine anhaltende Wirksamkeit von Dasatinib hinweist, selbst bei Patienten mit einer erheblichen Komorbiditätslast [24].

Bestimmte kardiovaskuläre und arterio-okklusive Ereignisse (AOEs) traten häufiger bei Patienten auf, die mit Dasatinib, Nilotinib und Bosutinib behandelt wurden, als bei mit Imatinib behandelten Patienten [15, 16, 25]. Dasatinib ist im Vergleich zu Imatinib mit einem erhöhten Risiko für die Entwicklung einer pulmonalen Hypertonie und einer kardialen Dysfunktion verbunden [5, 14]; Patienten mit Bluthochdruck, die mit Dasatinib behandelt werden, haben ein erhöhtes Risiko, Pleuraergüsse zu entwickeln [26, 27]. Nilotinib und Dasatinib können das QT-Intervall beeinflussen [4, 5], und insbesondere Nilotinib birgt im Vergleich zu Imatinib ein größeres Risiko für kardiovaskuläre und zerebrovaskuläre AOEs [18]. Unter Bosutinib wurde ein geringeres relatives AOE-Risiko beobachtet, das aber immer noch höher war als unter Imatinib [25]. Sobald ein 2G-TKI als beste Option angesehen wird, sind eine engmaschige Überwachung und ein aggressives Management von Komorbiditäten und anderen Risikofaktoren (z.B. Rauchen, Ernährung, Bewegungsmangel) wichtig, um das Risiko von AOEs zu minimieren.

Häufig sind Patienten mit CML übergewichtig, was oft mit kardiovaskulären Erkrankungen und Diabetes mellitus einhergeht [28]. Während Imatinib bei übergewichtigen Patienten eine verringerte Wirksamkeit gezeigt hat [29], ist das Ansprechen bei mit Dasatinib behandelten übergewichtigen Patienten vergleichbar zu Patienten mit Normalgewicht, wobei bei übergewichtigen Patienten eine überraschend schnellere mediane Zeit bis zum MMR erreicht wurde [30]; übergewichtige Patienten hatten jedoch ein höheres Risiko für einen Pleuraerguss als Patienten mit Normalgewicht (34% im Vergleich zu 25%) [30]. Übergewicht hatte keinen Einfluss auf das Ansprechen auf Bosutinib, doch übergewichtige Patienten, die mit Bosutinib behandelt wurden, wiesen im Vergleich zu Patienten mit Normalgewicht erhöhte Spiegel von Alanin- und Aspartat-Aminotransferase auf [31].

Bei der Auswahl von 2G-TKIs ist auch die Baseline-Überwachung auf Diabetes und Nieren-/Lebererkrankungen wichtig. Hyperglykämie und ein erhöhtes Risiko für die Entwicklung von Prädiabetes wurden mit der Behandlung mit Nilotinib in Verbindung gebracht [32], und Bosutinib und Nilotinib können eine Erhöhung der Alanin-Aminotransferase- und Lipase-Spiegel verursachen [4, 6]. Darüber hinaus wurden Bosutinib und Imatinib mit einer reversiblen Abnahme der glomerulären Filtrationsrate in Verbindung gebracht [33], obwohl dies in den meisten Fällen wahrscheinlich keine Nierenschädigung darstellt. Bei Patienten mit mehreren Komorbiditäten ist es wichtig, die potenziell erhöhten Risiken im Zusammenhang mit einem 2G-TKI sorgfältig abzuwägen und gleichzeitig sicherzustellen, dass der Patient das bestmögliche Therapieergebnis erzielen kann.

#### Überlegungen basierend auf Real-World-Evidenz: Kosten und Therapieadhärenz

Imatinib ist mittlerweile in mehreren Ländern als Generikum erhältlich. Der Zugang zu Dasatinib, Bosutinib und Nilotinib ist jedoch in vielen Ländern mit niedrigem und mittlerem Einkommen eingeschränkt, teilweise aufgrund von Regulierungsbehörden, die Imatinib als erste Wahl vorschreiben [34], oder aufgrund fehlender finanzieller Unterstützung für die Kostenübernahme von Markenmedikamenten. Darüber hinaus wirken sich die Kosten für Lohnausfälle und Reisen zu Arztterminen aus, selbst wenn die Kosten der TKIs durch Behandlungsunterstützungsprogramme subventioniert werden, insbesondere in Ländern mit niedrigem und mittlerem Einkommen [35].

Die Einhaltung der Behandlung ist entscheidend für die Verbesserung der Ansprechraten und des Überlebens. Bei Patienten, die mit Imatinib behandelt wurden, korrelierte eine Adhärenzrate von > 90% mit einer 6-Jahres-Wahrscheinlichkeit von 76% gegenüber 4% (P < 0,001), ein DMR (4-log-Reduktion von *BCR::ABL1* (MR^4^)) zu erreichen [36]. Real-World-Evidenz aus Ländern mit niedrigem und mittlerem Einkommen zeigte, dass eine verringerte Imatinib-Therapietreue mit einem geringeren ereignisfreien 10-Jahres-Überleben assoziiert war [37]. Eine erhöhte Adhärenz wurde auch mit reduzierten Krankenhauskosten und weniger Krankenhauseinweisungen in Verbindung gebracht [38, 39, 40, 41, 42].

Zahlreiche Faktoren beeinflussen die Adhärenz: UEs, Krankheits- und Behandlungsdauer, Vergessen der Medikamenteneinnahme, Unbequemlichkeit der Medikationshäufigkeit, Kosten, mangelndes Engagement und geringe krankheitsbezogene Aufklärung [43, 44, 45]. Eine verstärkte *BCR::ABL1*-Überwachung, niedrigere Zuzahlungen und weniger Tagesdosen korrelieren alle mit einer erhöhten Adhärenz [42, 45, 46]. Insgesamt sind die Aufklärung der Patienten über die Auswirkungen der Adhärenz und die regelmäßige Kommunikation zwischen Arzt und Patient in Bezug auf das UE-Management und finanzielle Fragen wichtig, um die Adhärenz zu optimieren. Die Überwachung der von den Patienten berichteten Behandlungsergebnisse kann dazu beitragen, frühe subtile Veränderungen zu erkennen, die sich auf die Therapietreue und/oder das allgemeine Wohlbefinden des Patienten auswirken können.

#### Zusammenfassung des Behandlungsansatzes

Der in Fallstudie 1 beschriebene Patient hat signifikante Komorbiditäten, die das Risiko erhöhen, AOEs zu entwickeln, die sorgfältig gegen seine Behandlungsziele abgewogen werden müssen. Angesichts der relativ hohen Überlebensraten kann Imatinib eine angemessene Wahl sein, wenn die Kosten für TKIs nicht durch staatliche Hilfsprogramme getragen werden. Wenn die Ziele des Patienten ein tieferes Ansprechen und das Erreichen einer TFR umfassen, wäre ein 2G-TKI ein besserer Kandidat.

Da dieser Patient ein hohes kardiovaskuläres Risiko hat, wäre der wahrscheinlich beste TKI in diesem Zusammenhang Bosutinib. Trotz der niedrigen Prozentsätze wurden kardiovaskuläre Ereignisse bei Nilotinib und Dasatinib im Vergleich zu Imatinib nach 5 Jahren häufiger berichtet [17, 18]. Wenn bei diesem Patienten bestimmte Lungenerkrankungen wie interstitielle Pneumonitis in der Vorgeschichte aufgetreten wären, sollte Dasatinib vermieden werden. Da dieser Patient eine Vorgeschichte von Diabetes mellitus hat, sollte Dasatinib aufgrund seines geringeren Risikos für metabolische Effekte im Vergleich zu Bosutinib oder Nilotinib in Betracht gezogen werden [4, 5, 6]. Die höhere Wahrscheinlichkeit einer anhaltenden MR^4,5^ unter Nilotinib im Vergleich zu Imatinib sollte gegen das Risiko kardiovaskulärer Ereignisse abgewogen werden.

AOEs sind selten tödlich, wie aus einer Studie hervorgeht, in der nur 3 Todesfälle mit Nilotinib (n = 563) und keiner mit Imatinib (n = 283) berichtet wurden [20]. Ohne ein angemessenes Management von Komorbiditäten ist die Wahrscheinlichkeit eines AOE und des Todes durch die Komorbiditäten möglicherweise höher als durch die CML. Ein aggressives Management der Komorbiditäten und die notwendigen Verhaltensänderungen (z.B. Ernährung, Bewegung) optimieren die Überlebensergebnisse bezüglich aller Ursachen. Dieser Patient sollte sich zu diesen Verhaltensänderungen bereit erklären, wenn er mit Bosutinib behandelt wird.

## Frühzeitige Umstellung von TKIs

Während der Behandlung werden die Patienten regelmäßig überwacht, um die *BCR::ABL1*-Transkriptspiegel als Reaktion auf die TKI-Therapie zu beurteilen. Das Erreichen eines EMR mit Imatinib und 2G-TKIs im 1L-Setting ist ein Prädiktor für ein DMR und ein verbessertes Überleben bei Patienten mit CML-CP. Daher ist es ein wichtiger Behandlungsmeilenstein. Wenn mit einem 1L-TKI kein EMR erreicht wird, kann ein Therapiewechsel in Betracht gezogen werden. Der Zeitpunkt des TKI-Wechsels bleibt jedoch ein kontroverses Thema, wobei jeder Kliniker seinen eigenen Ansatz hat. Eine regelmäßige Überwachung des Erstansprechens ist mit besseren Ergebnissen verbunden, da dadurch im Falle einer Unverträglichkeit/Resistenz ein sofortiger Wechsel gewährleistet ist. Eine frühere Umstellung auf 2G-TKI bietet die Hoffnung auf bessere Ergebnisse gegenüber einer späteren Umstellung bei Patienten, die bei der 1L-Behandlung kein EMR erreichen.

### Fallstudie 2

Eine 56-jährige Patientin mit CML mit mittlerem Risiko (beurteilt anhand des ELTS-Scores) wurde mit Imatinib (400 mg) behandelt und erreichte nach 3 Wochen ein hämatologisches Ansprechen. Während der Behandlung erlitt die Patientin einen Hautausschlag zweiten Grades und eine mäßige Flüssigkeitsretention; die Behandlung wurde für 10 Tage ausgesetzt, bis die Toxizitäten abgeklungen waren. Nach dem Abklingen der Toxizitäten nahm sie die Behandlung mit einer niedrigeren Imatinib-Dosis (300 mg) wieder auf. Eine Dosiserhöhung wurde versucht, aber es trat wieder eine mäßige Flüssigkeitsretention auf; daher wurde die Imatinib-Dosis bei 300 mg gehalten. Die Behandlungsbewertung nach 3 Monaten zeigte ein unzureichendes Ansprechen mit einem *BCR::ABL1*-Spiegel von 26%.

#### Überlegungen basierend auf klinischen Studien

Die ELN-Empfehlungen und NCCN-Richtlinien klassifizieren ein Therapieversagen als bestätigtes Fehlen eines EMR [11, 13]. Die Bestätigung ist besonders wichtig, wenn das zytogenetische Ansprechen nicht überwacht wird und/oder die *BCR::ABL1*-Spiegel nahe bei 10% liegen.

Eine Zweitlinien (2L)-Therapie mit Dasatinib und Nilotinib kann bei Patienten mit unzureichendem Ansprechen auf Imatinib zu hohen MMR-Raten führen [47]. Es zeigte sich, dass Patienten, die früh in der Behandlung ein tiefes Ansprechen erzielten, günstigere langfristige Ergebnisse zeigten als diejenigen, die später in der Behandlung ähnliche Ansprechraten erzielten, was die Bedeutung eines frühen gegenüber einem späten Wechsel hervorhebt [47, 48].

DASCERN war die erste prospektive Studie, die den potenziellen Nutzen einer frühen Umstellung auf Dasatinib bei Patienten zeigte, die nach 3-monatiger Behandlung mit Imatinib kein EMR erreichten [49]. Patienten, die nach 3 Monaten auf Dasatinib umgestellt wurden, hatten nach 12 Monaten eine signifikant höhere MMR-Rate als Patienten, die weiterhin Imatinib erhielten (29% im Vergleich zu 13%, P = 0,005), und kumulativ hatten bis Monat 24 mehr Patienten unter Dasatinib ein MMR erreicht (64% im Vergleich zu 41%), nachdem ein Behandlungswechsel erfolgt war [49]. Darüber hinaus deuteten die Ergebnisse der LASOR-Studie darauf hin, dass Patienten mit einem suboptimalen zytogenetischen Ansprechen (gemäß den weniger strengen ELN-Empfehlungen von 2009 [50]) nach 3 Monaten mit größerer Wahrscheinlichkeit ein verbessertes zytogenetisches und molekulares Ansprechen bei einer Umstellung auf Nilotinib als bei einer Eskalation der Imatinib-Dosis erreichten (CCyR nach 6 Monaten: 50% im Vergleich zu 36%, nominal P = 0,058), obwohl der Unterschied statistisch nicht signifikant war, wenn das Ansprechen nach der Umstellung mit einbezogen wurde [51].

Sobald ein Behandlungsversagen festgestellt wird, sinkt die Wahrscheinlichkeit, dass DMRs nach dem Wechsel zu einem 2G-TKI erreicht werden, während die Wahrscheinlichkeit einer Krankheitsprogression zunimmt. Bei Patienten aus den DASISION- und ENESTnd-Studien, die nach 3 Monaten kein EMR erreichten und eine Krankheitsprogression erlebten, verschlechterte sich der Krankheitszustand bei etwa der Hälfte zwischen 3 und 6 Monaten nach Feststellung des Behandlungsversagens [17, 52]. Die Ergebnisse dieser klinischen Studien liefern neue Einblicke in den potenziellen Nutzen einer Umstellung auf 2G-TKIs bei Patienten, die mit 1L-Imatinib kein EMR erreichen. Eine längere Nachbeobachtung ist jedoch gerechtfertigt, um festzustellen, ob ein früheres Ansprechen nach dem Wechsel zu einer Verbesserung der Überlebensergebnisse führen würde, was klinisch bedeutsamer sein könnte.

Real-World-Evidenz und praktische Erwägungen zur Überwachung des Ansprechens auf TKI

Die ELN-Empfehlungen besagen, dass eine *BCR::ABL1*-Mutationsanalyse durchgeführt werden muss, um die Behandlung mit dem wirksamsten TKI fortzusetzen [11]. Die retrospektive beobachtende TARGET-UK-Studie, die die Baseline-Monitoring-Praktiken bei britischen Patienten mit ND CML-CP bewertete, stellte jedoch fest, dass die ELN-Monitoring-Empfehlungen nicht konsequent umgesetzt wurden [53]. Dies führte bei Patienten zu einem höheren Rückfallrisiko: 23% der Patienten mit ELN-definiertem Behandlungsversagen wechselten die Behandlung nicht, und nur 49% der Patienten, die aufgrund eines Behandlungsversagens wechselten, hatten sich einer Mutationsanalyse unterzogen [53].

In der laufenden Beobachtungsstudie SIMPLICITY kam es bei 16% der Patienten innerhalb von 12 Monaten nach Beginn der Behandlung mit 1L-Imatinib, -Dasatinib oder -Nilotinib zu einem Behandlungswechsel [54]. Mehr Patienten wechselten zwischen 3 und 12 Monaten (69%) als innerhalb von 3 Monaten (31%), wobei die Umstellung bei Patienten, die mit Imatinib behandelt wurden, häufiger stattfand als bei mit Dasatinib oder Nilotinib behandelten Patienten [54, 55]. Primäre Gründe für einen Wechsel waren Intoleranz und Resistenz, beide häufiger bei Imatinib als bei Dasatinib (Unverträglichkeit: 42% im Vergleich zu 29%; Resistenz: 73% im Vergleich zu 14%) [54, 55].

Eine retrospektive Analyse der italienischen Arzneimittelbehörde (AIFA) zeigte, dass innerhalb des ersten Jahres der Behandlung mit 2G-TKIs 7% der Patienten die Behandlung wechselten (Dasatinib: 8%; Nilotinib: 7%). Über einen Zeitraum von 6 Jahren wechselten insgesamt 16% der mit Dasatinib und 11% der mit Nilotinib behandelten Patienten den TKI. Die Hauptgründe für einen Wechsel waren Intoleranz (59%) oder Resistenz (57%), wobei die meisten Patienten die Therapie innerhalb der ersten 12 Behandlungsmonate wechselten [56]. Es wurden keine spezifischen Baseline-Merkmale mit einer Unverträglichkeit in Verbindung gebracht, aber männliche Patienten schienen aufgrund von Resistenzen eher zu einem Behandlungswechsel zu neigen [56]. Ein Behandlungswechsel mit einem 1L-2G-TKI war relativ selten und trat in der Studie der AIFA viel seltener auf als in der SIMPLICITY-Studie [54, 55, 56]. Für die meisten Patienten mit Therapieversagen war Ponatinib die bevorzugte 2L-Option, aber die mediane Zeit bis zum Behandlungswechsel betrug 354 Tage. Insgesamt war die Wechselhäufigkeit in Real-World-Evidenzstudien geringer als in klinischen Studien [17, 18, 19], möglicherweise aufgrund von Studienprotokollen, die erfordern, dass die Patienten die Behandlung wechseln, sobald ein unzureichendes Ansprechen beobachtet wurde.

Bei Patienten, die mit einem 1L-2G-TKI behandelt werden und kein EMR haben, sollte eine Änderung der Behandlung mit Vorsicht erfolgen. Für Patienten, die aufgrund einer Unverträglichkeit eine Behandlungsänderung benötigen, könnte die Umstellung auf einen anderen 2G-TKI und/oder die Erwägung niedrigerer Behandlungsdosen die beste Option sein. Ponatinib scheint die bevorzugte nächste Behandlungsoption für Patienten mit Therapieversagen gegenüber Dasatinib, Nilotinib oder Bosutinib zu sein. Diese Empfehlung basiert jedoch auf Daten aus einem Setting, in dem Ponatinib in der dritten Therapielinie (3L) und darüber hinaus verwendet wurde. Zum Zeitpunkt der Erstellung dieses Übersichtsartikels liegen begrenzte prospektive Daten zur 2L-Therapie nach Resistenz gegen einen 2G-TKI vor, wobei eine aktuelle Beobachtungsstudie eine günstige Wirksamkeit bei der Verwendung von 2L-Ponatinib zeigte [57].

### Zusammenfassung des Behandlungsansatzes

Eine erfolgreiche Behandlung der CML kann eine sorgfältige Auswahl des anfänglichen TKI zusammen mit einer regelmäßigen Überwachung des Ansprechens und der Unverträglichkeit erfordern. Obwohl die Überwachung oft zu wenig genutzt wird, ist sie für fundierte Entscheidungen über Therapieänderungen wichtig, um das Progressionsrisiko nach fehlendem EMR bei der 1L-Therapie zu minimieren. Das Erkennen früher Anzeichen einer Unverträglichkeit oder eines Behandlungsversagens, gefolgt von einer frühzeitigen Umstellung, falls erforderlich, kann wichtig sein, um das beste Therapieergebnis für die Patienten zu gewährleisten.

Bei der in der Fallstudie 2 skizzierten Patientin ist ein Behandlungsversagen offensichtlich. Daher sollte die Behandlung rechtzeitig geändert werden, um das Risiko einer Krankheitsprogression zu minimieren und die Wahrscheinlichkeit optimaler Ergebnisse zu erhöhen. Da die Patientin mit 1L-Imatinib behandelt wurde, wäre ein Wechsel auf einen 2G-TKI angebracht. Liegen keine Kontraindikationen vor, würde Dasatinib basierend auf der DASCERN-Studie [49] empfohlen, wobei ein Wechsel auf Nilotinib basierend auf der LASOR-Studie [51] empfohlen wird, wenn die Patientin Dasatinib nicht verträgt. Aufgrund der Flüssigkeitsretention dieser Patientin in der Vorgeschichte und der Assoziation von Pleuraerguss mit Dasatinib wird eine Umstellung auf Dasatinib nicht empfohlen. Nilotinib könnte jedoch aufgrund der niedrigen Ödemraten, die zu diesem Mittel berichtet wurden, in Betracht gezogen werden [18]. Obwohl Ponatinib bei Patienten mit CML, die gegenüber 2G-TKIs resistent/intolerant sind, und bei Patienten mit T3151-Mutation Wirksamkeit gezeigt hat [58, 59], wird es allgemein für die Behandlung von CML bei diesen Patienten empfohlen [11, 13]. Da die Patientin in dieser Fallstudie keine T315I-Mutation trägt, empfehlen wir Ponatinib in diesem Falle nicht.

## Behandlungsfreie Remission

Die unbefristete Anwendung von TKIs ist ein üblicher erster Ansatz bei der Behandlung von Patienten mit CML, unabhängig vom Ansprechen [13, 50, 60]. Das Erreichen eines anhaltenden DMR während der Therapie wird nun als relevanter klinischer Endpunkt für Patienten angesehen, die letztendlich die Behandlung abbrechen möchten und dadurch eine TFR versuchen. Kollektive vorläufige Leitlinien besagen, dass Patienten, die mindestens 5 Jahre lang mit Imatinib oder 3 Jahre lang mit einem 2G-TKI behandelt wurden und mindestens 2 Jahre lang ein anhaltendes DMR erreichten (gemessen anhand einer Verringerung des *BCR::ABL1*-Spiegels (internationale Skala) auf < 0,01% (MR^4^), < 0,0032% (MR^4,5^) oder < 0,001% (5-log-Reduktion von *BCR::ABL1*) [11, 13]), während der TFR die TKI-Therapie einstellen können. Während der TFR ist eine regelmäßige Überwachung (monatlich in den ersten 6 Monaten, danach alle 2 Monate [11, 13]) der *BCR::ABL1*-Spiegel erforderlich, mit dem Ziel, ein sehr niedriges oder nicht nachweisbares Niveau der Resterkrankung aufrechtzuerhalten (Schwellenwert für einen Rückfall ist das MMR) [11, 13]. Weitere Untersuchungen sind erforderlich, um starke Prädiktoren für eine erfolgreiche TFR zu identifizieren.

Die für die TFR erforderliche Ansprechtiefe variierte in verschiedenen klinischen Studien, in denen die TFR untersucht wurde. In EURO-SKI war vor einem TFR-Versuch ein Mindestansprechen von MR^4^ erforderlich [61], aber die Wahrscheinlichkeit, behandlungsfrei zu bleiben, erschien bei strengeren Kriterien (STIM, TWISTER, A-STIM) höher und wurde mit einem stabileren Plateau in der Kurve der Ansprechrate assoziiert [62, 63, 64]. Darüber hinaus war eine längere DMR-Dauer vor dem TFR-Versuch mit einer geringeren Rückfallwahrscheinlichkeit verbunden [65].

### Fallstudie 3

Eine 33-jährige Patientin mit Niedrigrisiko-CML (wie anhand des ELTS-Scores bewertet) wurde mit 1L-Imatinib mit ausgezeichneter Verträglichkeit behandelt. Die Patientin erreichte nach 12 Behandlungsmonaten ein MMR, gefolgt von einem anhaltenden DMR über 4 Jahre. Sie möchte Imatinib absetzen, da sie erwägt, schwanger zu werden.

#### Erster TFR-Versuch (TFR1) - Vor- und Nachteile einer TFR

Eine Übersicht über die Ergebnisse der wichtigsten Studien zur TFR findet sich in Tabelle 2. Eine TFR nach anhaltendem DMR mit 1L-Imatinib wurde in den STIM- und A-STIM-Studien [62, 64] untersucht, in denen etwa 40–50% der Patienten in der Lage waren, die TFR für bis zu 7 oder mehr Jahre aufrechtzuerhalten. Patienten, die mit 2G-TKIs behandelt wurden, konnten ebenfalls eine TFR erreichen. In DASFREE (Tab 2), der bislang größten klinischen Studie zur Untersuchung der TFR bei Patienten, die Dasatinib in allen Therapielinien abgesetzt haben, waren 48% der Patienten, die Dasatinib abgesetzt hatten, nach 1 Jahr weiterhin in TFR, und die Remission war nach 2 Jahren dauerhaft (1 später Rückfall nach Monat 39) [66]. Patienten, die das MMR verloren und wieder mit Dasatinib begonnen hatten, sprachen schnell wieder an (die mediane Zeit bis zur Wiedererlangung des MMR und der MR^4,5^ betrug 2 bzw. 3 Monate) [66]. Darüber hinaus erwies sich das Absetzen von Dasatinib in der D-STOP-Studie (63% der Patienten behielten MR^4^ nach 1 Jahr) und der japanischen Phase-2-Studie zum Absetzen von Dasatinib (geschätzte 3-Jahres-TFR-Rate von 44%) als durchführbar [67, 68].

Eine erfolgreiche TFR wurde auch mit 1L- und 2L-Nilotinib nachgewiesen: 47% bzw. 48% der Patienten, die 1L- bzw. 2L-Nilotinib absetzten, blieben in den Studien ENESTfreedom und ENESTop nach 144 Wochen in TFR (Tab 2) [69, 70]. Darüber hinaus blieben in der STOP-2G-TKI-Studie, in der die TFR nach Absetzen von Dasatinib und Nilotinib überwacht wurde, 63% der Patienten nach 1 Jahr in TFR [71]. Obwohl das Rückfallrisiko in den ersten 6 Monaten am höchsten ist und nach 2 Jahren deutlich abnimmt, können späte Rückfälle auftreten; etwa 15% der Rezidive traten nach 2 Jahren auf [64], wobei Rezidive in der Blastenphase selten, aber möglich sind [72]. Die Beendigung der Behandlung bei TFR kann TKI-assoziierte UEs reduzieren, die Lebensqualität verbessern und die Behandlungskosten senken [61]. Das Absetzen der TKI-Therapie kann jedoch zu einem TKI-Entzugssyndrom (hauptsächlich muskuloskelettalen Schmerzen) führen, und die Patienten benötigen eine regelmäßige Überwachung der *BCR::ABL1*-Spiegel [11, 13, 66, 69, 70]. Darüber hinaus haben Studien, die die mit einer TFR verbundenen psychologischen Probleme untersuchten, gezeigt, dass aufgrund von Angst vor einem Rückfall oder der Verpflichtung zu einer regelmäßigen, häufigen *BCR::ABL1*-Überwachung nicht alle Patienten, die für einen TFR-Versuch infrage kamen, die Behandlung abbrechen wollten [73]. Auch die Verbesserungen der Lebensqualität waren in verschiedenen Studien bescheiden und widersprüchlich [74].

Unserer Erfahrung nach ist ein erheblicher Anteil der Patienten an einem Abbruch der Behandlung interessiert, und dies sollte von der Diagnose an diskutiert werden, wobei die TFR nur nach sorgfältiger Überlegung, Diskussion und Bewertung durch Kliniker versucht wird.

#### Erste TFR - Faktoren, die den Erfolg einer TFR beeinflussen

Eine längere Dauer der Imatinib-Behandlung vor der TFR war mit einem geringeren Rückfallrisiko verbunden [61, 62, 63]. Da die Behandlung mit 1L-2G-TKIs ein schnelleres und tieferes Ansprechen zeigt, ist es möglich, dass Patienten eine TFR nach einer kürzeren Exposition gegenüber 2G-TKIs als nach Imatinib versuchen können. In der ENESTfreedom-Studie hatten Patienten, die nach einer Behandlung mit Nilotinib für 3,5 Jahre eine TFR versuchten, ähnliche TFR-Raten wie Patienten, die länger als 6 Jahre mit Imatinib behandelt wurden [61, 62]. Die optimale DMR-Dauer vor dem Versuch einer TFR muss noch aufgeklärt werden, obwohl gezeigt wurde, dass eine verlängerte DMR-Dauer vor Beginn der TFR die Wahrscheinlichkeit erhöht, das MMR auch nach 6 Monaten aufrechtzuerhalten [61, 65].

Die Auswirkung des Sokal-Risikoscores auf den Erfolg der TFR wird derzeit untersucht; in den TWISTER- und STIM-1-Studien war jedoch ein höherer Sokal-Risikoscore mit einer niedrigeren TFR-Erfolgsrate verbunden [62, 63]. Andere Faktoren, die möglicherweise zum Erfolg der TFR beitragen, sind höheres Alter [66, 75], minimale Schwankungen der *BCR::ABL1*-Spiegel [64] und die Aufrechterhaltung der MR^4,5^ in den ersten 3 oder 4 Monaten nach der TFR [70, 76]. Fortschritte in der Methodik der Polymerase-Kettenreaktion können eine frühere Erkennung eines Rückfalls [77] und eine bessere Identifizierung geeigneter Patienten für eine TFR ermöglichen [78]. Eine Dosisreduktion vor dem TFR-Versuch (basierend auf der DESTINY-Studie) [79] kann das Risiko eines Entzugssyndroms verringern. Darüber hinaus deuten neuere Studien darauf hin, dass natürliche Killerzellen potenzielle Biomarker für die Vorhersage des Erfolgs der TFR sein können [80, 81].

Nicht alle Patienten kommen für eine TFR infrage: diejenigen, die eine Krankheitsprogression in die akute oder Blastenphase erlebt haben, selbst wenn sie seitdem zu CML-CP zurückgekehrt sind und das DMR wiedererlangt haben [12], außerdem diejenigen, die nicht häufig überwacht werden können, und diejenigen mit atypischen Transkripten, die nicht quantifiziert und daher ordnungsgemäß überwacht werden können. Das Erreichen einer TFR bei Patientinnen, die schwanger sind oder einen Schwangerschaftswunsch haben, bleibt ein kontroverses Thema. Einige Kliniker ziehen es vor, im Falle eines Rückfalls vor der Schwangerschaft eine TFR zu versuchen, während andere zu vorübergehenden Behandlungsunterbrechungen oder kompletten TFR-Versuchen während der Schwangerschaft neigen [11, 13]. Basierend auf der verfügbaren Evidenz wird für Patientinnen im gebärfähigen Alter eine Empfängnisverhütung empfohlen, und eine Schwangerschaft sollte erst geplant werden, nachdem ein stabiles Ansprechen erreicht ist [11]. Daher kann die TFR ein wichtiges Behandlungsziel für Patientinnen im gebärfähigen Alter sein.

Der Konsens über die TFR in der klinischen Praxis entwickelt sich noch, aber die Ergebnisse aus laufenden TFR-Studien werden mehr bestätigende Daten zu langfristigen Ergebnissen liefern. Bisher ist die TFR nur bei 20–30% der mit TKI behandelten Patienten erfolgreich [61, 62, 64, 66, 69, 70, 79]. Daher befinden sich weitere Ansätze zur Erhöhung der Zahl geeigneter Patienten und/oder zur Verringerung des Rückfallrisikos nach dem Absetzen, wie z.B. eine Kombinationstherapie, noch in der Entwicklung. Im Falle eines Rezidivs wird die Schwelle für die Wiederaufnahme der Behandlung noch untersucht. Frühe klinische Studien verwendeten die MR^4,5^ als Grenzwert für die Wiedereinführung der Behandlung [69], während spätere klinische Studien die MR^4^ oder sogar das MMR verwendeten [61, 62, 64, 66, 70]. Daten von ENESTfreedom zeigten, dass die meisten Patienten, die die MR^4^ verloren, nach weiterer Nachsorge auch das MMR verloren. Daher ist der Verlust des MMR oder der bestätigten MR^4^ ein angemessener Grenzwert für die Wiederaufnahme der Behandlung.

### Zusammenfassung des Behandlungsansatzes

Die Patientin in Fallstudie 3 ist eine Frau im gebärfähigen Alter, die mit 1L-Imatinib eine anhaltendes DMR erreicht hat und ein besonderes Interesse daran hat, die Behandlung abzubrechen; daher ist sie eine Kandidatin für eine TFR. Daten aus verschiedenen Studien (STIM, A-STIM, EURO-SKI, DESTINY) deuten darauf hin, dass mit Imatinib eine Wahrscheinlichkeit von etwa 50–60% besteht, die TFR bis zu 7 Jahre lang aufrechterhalten zu können (Tab 2) [61, 62, 64, 79]. Die Patientin sollte sich der Möglichkeit eines Rückfalls und der Notwendigkeit einer kontinuierlichen Überwachung während der TFR bewusst sein. Wenn jedoch ein Rückfall auftritt, kann das DMR nach erneutem Beginn der Behandlung mit Imatinib erfolgreich erreicht werden. Es sollte beachtet werden, dass eine Empfängnis den erneuten Behandlungsprozess erschweren würde und bei der Behandlungsentscheidung berücksichtigt werden sollte.

### Zweiter TFR-Versuch (TFR2)

Obwohl es gut dokumentierte Studien zu TFR1 gibt, sind nur begrenzte Daten zu TFR2 verfügbar. Wie in Tabelle 2 gezeigt, erleidet etwa die Hälfte der Patienten, die eine TFR versuchen, einen Rückfall, meistens innerhalb von 6 Monaten nach Absetzen der Behandlung [61, 62, 64, 66, 69, 70, 79]. In den meisten Fällen können die Patienten das DMR jedoch nach einer erneuten Behandlung wiedererlangen [66, 69, 70], was die TFR2 zu einer interessanten Diskussion für Kliniker macht.

Obwohl eine TFR2 möglich ist, haben die bisherigen Studien gemischte Ergebnisse geliefert. In der ReSTIM-Studie hatten 36% der Patienten eine erfolgreiche TFR2 nach Unterbrechung der Behandlung für im Median 5 Monaten [82]. In der TRAD-Studie blieben jedoch nur 22% der Patienten nach 6 Monaten in TFR2 [83]. In beiden Studien hatten Patienten, die innerhalb von 3 Monaten während der TFR1 einen Rückfall erlitten, mit größerer Wahrscheinlichkeit einen Rückfall während der TFR2. Ein bemerkenswerter Unterschied zwischen den beiden Studien ist die Dauer des DMR vor der TFR2. Eine längere DMR-Dauer vor der TFR2 kann in Betracht gezogen werden. Die Patienten sollten jedoch über eine geringere Wahrscheinlichkeit einer erfolgreichen TFR2 im Vergleich zur TFR1 informiert werden, und eine strenge Überwachung ist erforderlich. Im Falle eines TFR-Versagens bei Patienten, die mit 1L-Imatinib behandelt wurden, könnte der Wechsel zu einem 2G-TKI vor dem TFR2-Versuch eine von mehreren vernünftigen Strategien für Patienten mit einer tiefen Motivation für die TFR sein. Sollte die Patientin in Fallstudie 3 einen Rückfall erleiden, könnte daher ein 2G-TKI - obwohl nicht prospektiv getestet - bei der Wiederaufnahme der Behandlung in Betracht gezogen werden, um ein tieferes, dauerhafteres Ansprechen zu erreichen. Klinische Studien können eine Alternative sein.

Derzeit laufen klinische Studien zur Erforschung der Kombinationstherapie nach einem Rückfall aus der TFR1. Eine laufende Studie untersucht die Zugabe von Ruxolitinib zu verfügbaren TKIs der ersten/zweiten Generation nach einem Rückfall nach der TFR1 mit dem Ziel, die Wahrscheinlichkeit einer erfolgreichen TFR2 zu erhöhen (NCT03610971). Eine ähnliche Studie zur Untersuchung der Zugabe von Asciminib zu Imatinib bei Patienten, die mit Imatinib behandelt wurden und einen Rückfall nach TFR1 erlitten hatten, läuft (NCT04838041).

## COVID-19

Die COVID-19-Pandemie hat die klinische Praxis, Überwachung und Behandlung von Krebs im Allgemeinen, einschließlich CML, stark beeinflusst. Aufgrund von Präventivmaßnahmen kann der Zugang zur Klinik eingeschränkt oder für die Fernversorgung angepasst sein, was bedeutet, dass Patienten die Klinik möglicherweise nicht regelmäßig besuchen und eventuell modifizierte Methoden zur Diagnose benötigen. Somit besteht die Gefahr, dass mangelndes Ansprechen und/oder eine Unverträglichkeit verspätet erkannt werden oder im schlimmsten Fall die Behandlung verzögert wird, bis sich die Krankheit in einem fortgeschritteneren Stadium befindet. Darüber hinaus können Patienten, die eine TFR versuchen, Schwierigkeiten haben, Termine zur regelmäßigen Überwachung wahrzunehmen, was den Zeitpunkt für die Wiederaufnahme der Behandlung verzögern und das Risiko eines erneuten Auftretens/einer Progression erhöhen kann. Trotz Hinweisen aus Vorstudien [84] gibt es bisher keine Evidenz dafür, dass eine TKI-Therapie eine schützende Wirkung für Patienten mit CML vor einer SARS-CoV-2-Infektion haben oder die Behandlungsergebnisse für Patienten, die mit SARS-CoV-2 infiziert sind, verschlechtern kann.

Die American Society of Hematology und die International CML Foundation haben eine Reihe von Leitlinien für Patienten und Kliniker herausgegeben, die auf weltweiten Erfahrungen basieren [85, 86]. Patienten mit ND CML-CP sollten gemäß dem Standardprotokoll überwacht und behandelt werden, und Patienten mit CML-CP, die sich bereits einer TKI-Therapie unterziehen, sollten ihr aktuelles Regime fortsetzen. Im Falle einer Infektion mit SARS-CoV-2 sollte die TKI-Therapie fortgesetzt werden. Um das Infektionsrisiko mit SARS-CoV-2 zu minimieren, sollte die *BCR::ABL1*-Überwachung der Patienten nach Möglichkeit aus der Ferne über Probenentnahmekits für zu Hause erfolgen.

Bisher empfehlen die Leitlinien die Impfung gegen COVID-19 nach Rücksprache mit dem Gesundheitsteam des Patienten. Im Allgemeinen sind Patienten mit CML wohl nicht immungeschwächt, und die verfügbaren Daten deuten auf eine gute Immunantwort auf die COVID-19-Impfstoffe hin. Expertenempfehlungen wurden an anderer Stelle veröffentlicht [87].

## Neue/zukünftige Behandlungsansätze

Neue Behandlungen werden für stark vorbehandelte Patienten und für diejenigen entwickelt, die intolerant sind oder bei zugelassenen Therapien eine Resistenz oder Krankheitsprogression erfahren haben. Die US-amerikanische Food and Drug Administration hat kürzlich Asciminib zugelassen, einen neuartigen STAMP-Inhibitor, der Erste seiner Klasse, der gegen die multi-TKI-resistente T315I-Mutation wirksam ist [88].

Die Wirksamkeit von Asciminib bei Patienten mit > 2 vorherigen TKI wurde in der Phase-3-Studie ASCEMBL [89] gezeigt. Patienten, die mit Asciminib (2 Dosen von 40 mg pro Tag) behandelt wurden, zeigten nach 24 Wochen eine statistisch signifikante Verbesserung des MMR im Vergleich zu Bosutinib (25,5% im Vergleich zu 13,2%, 2-seitig P = 0,029), wobei Thrombozytopenie und Neutropenie die häufigsten Nebenwirkungen im Zusammenhang mit Asciminib sind. Auch eine Hypertonie wurde bei mit Asciminib behandelten Patienten häufiger beobachtet als bei Patienten, die mit Bosutinib behandelt wurden (11,5% im Vergleich zu 3,9%), und bei 5 Patienten (3,2%), die mit Asciminib behandelt wurden, kam es zu AOEs (2 mit tödlichem Ausgang) im Vergleich zu 1 Patienten, der mit Bosutinib behandelt wurde (1,3%) [89]. Mutationen, die Resistenz gegen Asciminib verleihen, entwickelten sich selten während der In-vivo-Tests. Darüber hinaus hat sich Asciminib in Kombination mit TKIs, die auf die ATPase-Domäne von *BCR::ABL1* abzielen, aufgrund seines ausgeprägten Wirkmechanismus, der auf die Myristoyl-Tasche abzielt, als hilfreich erwiesen, um die Entstehung von Resistenzen zu unterdrücken [90, 91, 92], was Anlass zu weiteren Untersuchungen gibt. Außerdem scheint Asciminib als Monotherapie gegen bestimmte *BCR::ABL*^T315I^- und *BCR::ABL*^F359I^-Mutationen nicht wirksam zu sein. Daher kann bei einigen Patienten eine Kombinationstherapie erforderlich sein [90]. Ein Vergleich zwischen Asciminib und Ponatinib wäre von großem Interesse.

Obwohl es sich nicht um einen neuen Wirkstoff handelt, wurde für Ponatinib in der OPTIC-Studie [93] ein angepasstes Verabreichungsschema (auf das Ansprechen gerichtete Dosisreduktion) verwendet, das das Risiko von AOEs verringern kann. Dieser an das Ansprechen angepasste Ansatz kann in Zukunft für die Gesamtbehandlung mit TKI in Betracht gezogen werden.

Darüber hinaus gibt es weitere potenzielle Wirkstoffe für Patienten, bei denen ein Therapieversagen oder eine Unverträglichkeit gegenüber Dasatinib, Nilotinib oder Bosutinib auftritt (Tab 3). Dazu gehören die 2G-TKIs Radotinib und Flumatinib, die beide in klinischen Phase-3-Studien mit ND CML-CP eine verbesserte Wirksamkeit gegenüber Imatinib mit tolerierbaren Sicherheitsprofilen gezeigt haben [94, 95] und als potenzielle 2L-Optionen für CML-CP-Patienten, die resistent oder intolerant gegenüber einer 1L-Therapie sind, geprüft werden. Dazu gehören auch die TKIs der dritten Generation Vodobatinib und Olver-Embatinib [96, 97, 98, 99] und PF-114, ein potenter TKI, der seine Wirksamkeit in einer Phase-1-Studie bei Patienten mit CML-CP gezeigt hat, die zuvor mit mindestens 2 Therapien behandelt wurden, oder bei Patienten mit der T315I-Mutation, die > 6 Monate behandelt wurden [100]. Das kontinuierliche Aufkommen neuer Therapien wird begrüßt und wird die Art und Weise, wie Kliniker CML behandeln, in Zukunft verändern.

## Schlussfolgerungen

Da mehr zugelassene TKIs zur Verfügung stehen, sind die Behandlungsentscheidungen komplexer geworden. Die Wahl der Behandlung im 1L-Setting wird nicht nur von der Wirksamkeit und Sicherheit der TKIs beeinflusst, sondern auch von patientenspezifischen Faktoren und praktischen Erwägungen. Auch der Patientenwunsch und die persönlichen Umstände wirken sich zunehmend auf die Behandlungsstrategien aus. Es hat sich gezeigt, dass eine regelmäßige Überwachung zur Information über Behandlungsoptionen im Falle eines Therapieversagens/einer Unverträglichkeit gegenüber der 1L-Therapie und ein frühzeitiger Wechsel das Ansprechen der Patienten verbessern. Die verbesserte Wirksamkeit mit 2G-TKIs hat zu einer erhöhten Wahrscheinlichkeit geführt, ein DMR zu erreichen. Daher wird eine TFR schnell zu einem Behandlungsziel für Patienten. Mehr mit 2G-TKI behandelte Patienten erreichen eine TFR als Patienten mit Imatinib. In den meisten Fällen blieben Patienten mit Rückfall empfindlich gegenüber TKIs und erreichten bei erneuter Behandlung wieder ein MMR. Da Patienten mit TFR einen Rückfall erleiden können, ist ein besseres Verständnis einer zweiten TFR wichtig, um fundierte Behandlungsentscheidungen zu treffen. Darüber hinaus war die Behandlung von CML während der COVID-19-Pandemie eine Herausforderung, aber die Veröffentlichung von Richtlinien und Empfehlungen zur Fortsetzung der Behandlung und zur Impfung hat zur Unterstützung der Ärzte und Patienten beigetragen. Schließlich erweitert das jüngste Aufkommen neuer Therapien die Behandlungsoptionen für Patienten mit CML, insbesondere für diejenigen mit der T315I-Mutation.

## Abkürzungen und Akronyme

1G = erste Generation, Erstgenerations-; 1L = Erstlinie; 2G = zweite Generation, Zweitgenerations-; 2L = Zweitlinie; 3G = dritte Generation, Drittgenerations-; 4G = vierte Generation, Viertgenerations-; UE = unerwünschtes Ereignis; AOE = arteriell-okklusives Ereignis; AP = beschleunigte Phase; BID = zweimal täglich; CCI = Charlson Comorbidity Index; CCyR = komplettes zytogenetisches Ansprechen; CHR = komplettes hämatologisches Ansprechen; CML = chronische myeloische Leukämie; CML-CP = chronische myeloische Leukämie in der chronischen Phase; CMR = komplettes medizinisches Ansprechen; DMR = tiefes molekulares Ansprechen; ECOG PS = Eastern Cooperative Oncology Group Performance Status; ELN = Europäisches LeukämieNet; ELTS = European Treatment and Outcome Study Long-Term Survival; EMR = frühes molekulares Ansprechen; MCyR = gutes zytogenetisches Ansprechen; MMR = gutes molekulares Ansprechen; MR^4^ = 4-log-Reduktion des *BCR::ABL1*-Spiegels; MR^4,5^ = 4,5-log-Reduktion des *BCR::ABL1*-Spiegels; mRFS = molekulares rezidivfreies Überleben; MTD = maximal tolerierte Dosis; NCCN = National Comprehensive Cancer Network; ND = neu diagnostiziert; Ph+ = Philadelphia-Chromosom-positiv; TEAE = bei der Behandlung auftretendes unerwünschtes Ereignis; TFR = behandlungsfreie Remission; TFR1 = erste behandlungsfreie Remission (Versuch 1); TFR2 = zweite behandlungsfreie Remission (Versuch 2); TKI = Tyrosinkinase-Inhibitor; TRAE = behandlungsbedingtes unerwünschtes Ereignis.

## Danksagung

Schreib- und Redaktionsunterstützung wurden von Flint Stevenson-Jones, PhD, Caudex bereitgestellt, und von Bristol Myers Squibb finanziert.

## Beiträge der Autoren

Alle Autoren haben zur Entwicklung des Manuskripts beigetragen und den endgültigen Entwurf zur Veröffentlichung freigegeben.

## Finanzierung

Die Finanzierung erfolgte durch Bristol Myers Squibb.

## Verfügbarkeit von Daten und Materialien

Nicht zutreffend.

## Erklärungen

Zustimmung der Ethikkommission und Einwilligung in die Teilnahme

Nicht zutreffend.

## Zustimmung zur Veröffentlichung

Nicht zutreffend.

## Interessenkonflikte

VGG - Förderung: Bristol Myers Squibb, Incyte, Novartis, Pfizer. Beratungshonorare: Bristol Myers Squibb, Incyte, Novartis, Pfizer. Zahlung/Honorare: Bristol Myers Squibb, Incyte, Novartis, Pfizer. Reiseunterstützung: Bristol Myers Squibb, Incyte, Novartis, Pfizer. Datenschutzüberwachungsausschuss oder Beirat: Bristol Myers Squibb, Incyte, Novartis, Pfizer. Leitung: Vorstand von GELMC. MB - Beratertätigkeit: Bristol Myers Squibb/Celgene, Incyte, Novartis, Pfizer. EJ - Forschungsgelder: AbbVie, Amgen, Bristol Myers Squibb, Pfizer, Takeda. Beratertätigkeit: AbbVie, Amgen, Bristol Myers Squibb, Genentech, Novartis, Pfizer, Takeda. MM - Förderung: Bristol Myers Squibb, Novartis, Sun Pharma/SPARC. Beratungshonorare: Bristol Myers Squibb, Novartis, Pfizer, Takeda. Zahlung/Honorar: Bristol Myers Squibb, Novartis, Pfizer, Takeda. Reiseunterstützung: Bristol Myers Squibb, Novartis, Pfizer, Takeda. JC - Förderung: Bristol Myers Squibb, Novartis, Pfizer, Sun Pharma, Takeda. Beratertätigkeit: Novartis, Pfizer, Sun Pharma, Takeda.

Eingereicht: 10. März 2022; angenommen: 2. Juni 2022

Online publiziert: 11. Juli 2022

## Lizenzangabe

© Der/die Autor(en) 2022. Open Access: Dieser Artikel wird gemäß den Bedingungen der Creative Commons Attribution 4.0 International License lizenziert. Diese Lizenz erlaubt eine uneingeschränkte Verwendung, Vervielfältigung, Verbreitung, Bearbeitung und Reproduktion in jedem Medium oder Format, sofern die ursprünglichen Autoren und die Quelle in angemessener Weise genannt werden, ein Link zur Creative-Commons-Lizenz angegeben und dargelegt wird, ob Änderungen vorgenommen wurden. Die Bilder oder andere Materialien von Drittanbietern in diesem Artikel sind in der Creative-Commons-Lizenz des Artikels enthalten, sofern in einer Quellenangabe für das Material nichts anderes angegeben ist. Wenn das Material nicht in der Creative-Commons-Lizenz des Artikels enthalten ist und die von Ihnen beabsichtigte Verwendung aufgrund gesetzlicher Bestimmungen nicht gestattet ist oder über die erlaubte Verwendung hinausgeht, müssen Sie die Erlaubnis direkt beim Inhaber der Urheberrechte einholen. Um eine Kopie dieser Lizenz anzuzeigen, besuchen Sie *httpl/creativecommons.org/licenses/by/4.0/*. Für die in diesem Artikel zur Verfügung gestellten Daten gilt die Verzichtserklärung der Creative Commons Public Domain.

## Figures and Tables

**Tab. 1:** Wirksamkeit von 2G-TKIs bei neu diagnostizierter CML-CP

Studie	Einschlusskriterien	Wichtige Wirksamkeitsdaten	Sicherheit	Behandlungsabbrüche und Gründe
*Dasatinib.* DAS1SION (NCT00481247) [17]: multizentrische Phase-3-Studie zum Vergleich von Dasatinib mit Imatinib in der 1L-Behandlung von ND Ph+ CML-CP	Alter > 18 Jahre; neu diagnostizierte Ph+ CML-CP; ECOG PS 0–2; keine vorherige TKI-Behandlung* ; kein Pleuraerguss zu Beginn. Ausgewählte kardiovaskuläre Erkrankungen, die nicht ausgeschlossen sind: Myokardinfarkt > 6 Monate, Herzinsuffizienz > 3 Monate oder unkontrollierte Angina pectoris > 3 Monate vor der Teilnahme	Bestätigtes CCyR nach 12 Monate (primärer Endpunkt): Dasatinib 77%, Imatinib 66% (P = 0,007); MMR nach 12 Monaten: Dasatinib 46%, Imatinib 28% (P = 0,0001); kumulative 5-Jahres-MMR-Rate: Dasatinib 76%, Imatinib 64% (P = 0,002); kumulative 5-Jahres-MR^4,5^-Rate: Dasatinib 42%; Imatinib 33% (P = 0,0251)	Medikamentenbedingter Pleuraerguss: Dasatinib 28%, Imatinib 1%	Abbrüche: Dasatinib (n = 100, 39%), Imatinib (n = 96, 37%); Dasatinib: Unverträglichkeit (n = 42, 16%), Progression oder Therapieversagen (n = 28, 11%); Imatinib: Progression oder Therapieversagen (n = 36, 14%), Unverträglichkeit (n = 17, 7%)

*Nilotinib.* ENESTnd (NCT00471497) [18]: randomisierte, offene, multizentrische Phase-3-Studie mit Nilotinib (300 mg oder 400 mg BID) versus Imatinib bei Patienten mit ND Ph+ CML-CP	Alter > 18 Jahre: Ph+ CML-CP innerhalb von 6 Monaten nach der Diagnose diagnostiziert; ECOG PS 0–2; keine Herz-Kreislauf-Erkrankungen; keine T315I-Mutationen	MMR nach 12 Monaten (primärer Endpunkt): Nilotinib 300 mg 44%, Nilotinib 400 mg 43%, Imatinib 22% (P < 0,001 Nilotinib im Vergleich zu Imatinib); 5-Jahres-MR^4,5^: Nilotinib 300 mg 54% (n = 151/279), Nilotinib 400 mg 52% (n = 147/277), Imatinib 31% (n = 89/280) (P < 0,0001 Nilotinib im Vergleich Imatinib); geschätztes progressionsfreies Überleben: Nilotinib 300 mg 96%, Imatinib 91% (P = 0,0204); geschätztes Gesamtüberleben: Nilotinib 300 mg 96%, Imatinib 92% (P = 0,0266)	Kardiovaskuläre Ereignisse Grad 3/4: Nilotinib 300 mg 5% (n = 13/279), Nilotinib 400 mg 9% (n = 24/277), Imatinib 2% (n = 5/280); Grad 3/4 erhöhte Glukosespiegel: Nilotinib 300 mg 7% (n = 20/279), Nilotinib 400 mg 7% (n = 19/277), Imatinib < 1% (n = 1/280)	Absetzen von Imatinib (n = 139): suboptimales Ansprechen/Behandlungsversagen (n = 59), UE/ auffällige Laborwerte (n = 38); Absetzen von Nilotinib 300 mg (n = 110): UE/auffällige Laborwerte (n = 34), suboptimales Ansprechen/ Behandlungsversagen (n = 34); Abbrüche von Nilotinib 400 mg (n = 105): UE/auffällige Laborwerte (n = 56), Widerruf der Einwilligung (n = 16), suboptimales Ansprechen/Therapieversagen (n = 13)

*Bosutinib.* BFORE (NCT02130557) [15, 25, 101, 102]: randomisierte, offene, multizentrische Phase-3-Studie zu Bosutinib im Vergleich zu Imatinib bei Patienten mit ND Ph+ oder *Ph−/BCR::ABL1+* CML-CP	Alter > 18 Jahre; neu diagnostizierter Ph+ oder *Ph−/BCR::ABL1+* CML-CP (< 6 Monate nach der Erstdiagnose); ECOG PS 0–1; keine vorherige Behandlung, einschließlich TKIs	MMR nach 12 Monaten (primärer Endpunkt): Bosutinib 47%; Imatinib 37% (P = 0,0200); CCyR nach 12 Monaten: Bosutinib 77%, Imatinib 66% (P = 0,0075)	Grad > 3 vaskuläre Ereignisse nach 18 Monaten: Bosutinib 2%, Imatinib 0%; die häufigsten nichthämatologischen TEAEs Grad > 3: erhöhte Alanin-Aminotransaminase: Bosutinib 21%, Imatinib 2%; erhöhte Aspartat-Aminotransferase: Bosutinib 10%, Imatinib 2%	Absetzen von Bosutinib nach 12 Monaten (n = 59/268, 22%): UE (n = 37, 14%), Patientenwunsch (n = 6,2%); Imatinib-Abbrüche nach 12 Monaten (n = 71/265, 27%): UE (n = 24, 9%), suboptimales Ansprechen/Therapieversagen (n = 16, 6%)

*Nilotinib und Dasatinib.* JALSG CML212 (#UMIN000007909) [103]: Phase 3, randomisierte, offene, multizentrische Studie zum Erreichen einer MR^4,5^ nach Behandlung mit Nilotinib im Vergleich zu Dasatinib	Neu diagnostizierte CML-CP, bestätigt durch zytogenetische Untersuchung und/oder Nachweis von *BCR::ABL1* durch RT-PCR	Kumulative MR^4,5^-Raten nach 18 Monaten (primärer Endpunkt): Nilotinib 33%, Dasatinib 31%	UE-Grad 3/4 mit einer Häufigkeit von > 10%; Nilotinib: Erhöhung der Lipase (12%), UE-Grad 3/4 mit einer Häufigkeit von > 10%; Dasatinib: Thrombozytopenie (17%), Neutropenie (13%)	Abbruch der Behandlung nach 18 Monaten: 24% der mit Nilotinib und 20% der mit Dasatinib behandelten Patienten

2G = zweite Generation, UE = unerwünschtes Ereignis, CCyR = komplettes zytogenetisches Ansprechen, CML-CP = chronische myeloische Leukämie in der chronischen Phase, ECOG PS = Eastern Cooperative Oncology Group Performance Status, MMR = gutes molekulares Ansprechen, MR^4,5^ = 4,5-log-Reduktion des *BCR::ABL1*-Spiegels, ND = neu diagnostiziert, Ph+ = Philadelphia-Chrpmosom-positiv; TEAE = bei der Behandlung auftretendes unerwünschtes Ereignis.

* Vorherige TKI ermöglichten das erforderliche Krankheitsmanagement während des Wartens auf den Studienbeginn; kommerziell erhältliche Liefermengen von Gleevec (Glivec) in jeder Dosis könnten verschrieben werden, aber für nicht länger als 2 Wochen Dauer.

**Tab. 2:** Zusammenfassung klinischer Studien zur Untersuchung der behandlungsfreien Remission

Studie	Patientenpopulation	Schlüsseldaten zur Wirksamkeit	Schlüsseldaten zur Sicherheit
*Dasatinib DASFREE* (NCT01850004) [66]: Phase-2-Studie zum Absetzen der Dasatinib-Therapie bei Patienten mit CML-CP und stabiler MR^4,5^	Alter > 18 Jahre; Dasatinib-Behandlung für > 2 Jahre als 1L- oder nachfolgende CML-CP-Therapie; Dasatinib-induziertes DMR (MR^4,5^) für > 1 Jahr vor Teilnahme; ECOG PS 0–1	TFR nach 12 Monaten (primärer Endpunkt), insgesamt: 48%, Abbruch nach 1L-Dasatinib: 54%, Abbruch nach 2L + Dasatinib: 43%; MMR nach 24 Monaten (sekundärer Endpunkt): 45%	Bei 11% der Patienten (n = 9) kam es zu vom Prüfarzt festgestellten Absetzereignissen, einschließlich muskuloskelettaler Schmerzen und arterieller Hypertonie

*Nilotinib ENESTop* (NCT01698905) [69]: Absetzen von Nilotinib bei Patienten mit *BCR::ABL+* CML-CP, die mit Nilotinib nach Imatinib eine anhaltende MR^4,5^ erreicht haben	Alter > 18 Jahre; 2L-Nilotinib für > 2 Jahre nach Imatinib; *BCR::ABL+* CML-CP; keine MR^4,5^ unter Imatinib; MR^4,5^ erreicht unter Nilotinib; ECOG PS 0–2	TFR (MMR) nach 48 Wochen (primärer Endpunkt): 52%	Muskuloskelettale Schmerzen in den ersten 48 Wochen, berichtet von 34% der Patienten

*Nilotinib ENESTfreedom* (NCT01784068) [70]: Absetzen von Nilotinib bei Patienten mit b3a2/b2a2 CML-CP, die mit 1L-Nilotinib ein anhaltendes DMR erreicht haben	Alter > 18 Jahre; 1L-Nilotinib für > 2 Jahre; CML-CP mit b3a2 und/oder b2a2; MR^4,5^ beim Screening; ECOG PS 0–2	TFR (MMR) nach 48 Wochen (primärer Endpunkt): 52%	Muskuloskelettale Schmerzen in den ersten 48 Wochen, berichtet von 34% der Patienten

*Nilotinib ENESTgoal* (NCT01744665) [104]: Absetzen von Nilotinib bei Patienten mit CML-CP, die nach Umstellung auf Nilotinib eine anhaltende MR^4,5^ erreicht haben	1L-Imatinib für > 1 Jahr; CML-CP mit MMR aber nicht MR^4,5^; qualitative Polymerase-Kettenreaktion in Echtzeit alle 3 Monate	mRFS nach 6 Monaten: 7/17 Patienten erhielten MR^4,5^ aufrecht	18% der Patienten erlitten UEs während der TFR

*Imatinib STIM* (NCT00478987) [62]: Beobachtungsstudie zur CMR-Persistenz nach Absetzen der Imatinib-Therapie	Alter > 18 Jahre; CML-CP; CMR unter Behandlung mit Imatinib für > 2 Jahre; keine vorherigen Behandlungen: immunmodulatorisch (außer Interferon-α), autologe hämatopoetische Stammzelltransplantation oder für andere bösartige Erkrankungen	TFR: 6 Monate: 43%; 60 Monate: 38%	Es wurden keine muskuloskelettalen Schmerzen berichtet; 1 Patient entwickelte nach einem Rückfall und Wiederaufnahme der TKI-Behandlung eine lymphatische Blastenkrise

*Imatinib A-STIM* (NCT02897245) [64]: Beobachtungsstudie zur MMR-Persistenz nach Absetzen der Imatinib-Therapie	CML-CP; Behandlung mit Imatinib; CMR in Behandlung; DMR (*BCR::ABL1* internationale Skala < 0,01%)	TFR ohne Verlust der MMR (primäres Ergebnis): 1 Jahr: 57%; 3 Jahre: 53%; 5 Jahre: 51%; 7 Jahre: 46%	Keine Sicherheitssignale gemeldet

*1L-TKI-EURO-SKI* (NCT01596114) [61]: Phase 3, multizentrische, offene Studie zur Bewertung der Persistenz einer MR bei Patienten mit CML nach Absetzen von TKI	Alter > 18 Jahre; CML-CP mit 1L-TKI-Behandlung oder 2L bei Umstellung aufgrund der Toxizität von 1L-TKI; > 3 Jahre vorherige TKI-Therapie; > MR^4^ für > 1 Jahr	mRFS (primärer Endpunkt): 6 Monate: 61%; 24 Monate: 50%; 49% der Patienten verloren das MMR nach Absetzen von TKI	4 Todesfälle ohne Bezug zur CML; jeweils 1 Myokardinfarkt, Lungenkrebs, Nierenkrebs und Herzinsuffizienz; 6 Todesfälle ohne Zusammenhanf mit CML-CP nach Verlust des MMR und Wiederaufnahme der Behandlung

*L-TKI-DESTINY* (NCT01804985) [79]: Großbritannien, Phase 2, offene, multizentrische Studie zur TKI-Deeskalation und zum Absetzen bei Patienten mit ausgezeichnetem Ansprechen auf die TKI-Behandlung	Alter > 18 Jahre; *BCR::ABL1*+ CML-CP; > 3 Jahre vorherige TKI-Therapie; > 3 qualitative Polymerase-Kettenreaktion; Transkripte von < 0,1% *BCR::ABL1* in den 12 Monaten vor der Teilnahme	Rückfallfreies Überleben nach 12 Monaten Deeskalation und 2 Jahren Behandlungsabbruch (primärer Endpunkt); Patienten mit MR^4^ bei Studieneintritt: 72%; Patienten mit MMR bei Studieneintritt: 36%	2 Todesfälle aufgrund nicht zusammenhängender Ursachen

1L = Erstlinie, 2L = Zweitlinie, UE = unerwünschtes Ereignis, CCyR = komplettes zytogenetisches Ansprechen, CML-CP = chronische myeloische Leukämie in der chronischen Phase, CMR = komplettes molekulares Ansprechen, ECOG PS = Eastern Cooperative Oncology Group Performance Status, EMR = frühes molekulares Ansprechen, MMR = gutes molekulares Ansprechen, MR^4^ = 4-log-Reduktion von *BCR::ABL1,* MR^4,5^ = 4,5-log-Reduktion von *BCR::ABL1,* mRFS molekulares rezidivfreies Überleben, TFR = behandlungsfreie Remission, TKI = Tyrosinkinase-Inhibitor.

**Tab. 3:** Zusammenfassung der zukünftigen Behandlungslandschaft

Wichtige Studieninformationen	Wichtige Daten zur Wirksamkeit	Wichtige Daten zur Sicherheit
*2G-TK1sRadotinib:* 2G-TKI mit Aktivität gegen natives und Kinasedomänen-mutiertes *BCR:ABL,* wird derzeit getestet, um die Wirksamkeit bei CML-CP nach Versagen oder Unverträglichkeit gegenüber einer vorherigen TKI-Therapie zu bewerten [105]		

*RERISE* (NCT01511289) [106]: Phase-3-Studie, vergleicht Radotinib mit Imatinib bei Patienten mit ND CML-CP in der Republik Korea, Indonesien, den Philippinen und Thailand	MMR nach 12 Monaten (primärer Endpunkt): Radotinib 300 mg BID 52% (P = 0,0044 im Vergleich zu Imatinib), Imatinib 30%; CCyR nach 12 Monaten (sekundärer Endpunkt): Radotinib 300 mg BID 91% (P = 0,0120 im Vergleich zu Imatinib), Radotinib 400 mg BID 82% (nicht signifikant im Vergleich zu Imatinib), Imatinib 77%	Grad 3–4 Neutropenie war am häufigsten berichtetes hämatologisches UE: Radotinib 300 mg 19%, Radotinib 400 mg 23%, Imatinib 32%

Phase 3, multinationale (Republik Korea, Türkei, Russische Föderation und Ukraine) Studie zur Beurteilung der Wirksamkeit bei CML-CP nach Versagen oder Unverträglichkeit gegenüber einer vorherigen TKI-Therapie (NCT03459534; rekrutiert derzeit)	Daten noch nicht verfügbar	Daten noch nicht verfügbar

*Flumatinib:* Imatinib-Derivat, das eine erhöhte Wirksamkeit gegenüber Imatinib bei chinesischen Patienten mit ND CML-CP mit einem ähnlichen Sicherheitsprofil zeigt [95]		

FESTnd (NCT02204644) [95]: Phase-3-Studie: Flumatinib im Vergleich zu Imatinib bei ND CML-CP	MMR nach 6 Monaten (primärer Endpunkt): Flumatinib 34%, Imatinib 18% (P = 0,0006); EMR nach 3 Monaten (sekundärer Endpunkt): Flumatinib 82%, Imatinib 53% (P < 0,0001)	Nebenwirkungen aller Schweregrade häufiger im Flumatinib-Arm: Diarrhoe (n = 79/196, 40%), Alanin-Transaminase-Erhöhung (n = 51/196, 26%); Nebenwirkungen aller Schweregrade häufiger im Imatinib-Arm: Ödem (n = 70/198, 35%), Schmerzen in den Extremitäten (n = 49/198, 25%), Hautausschlag (n = 28/198, 14%), Neutropenie, Thrombozytopenie, Anämie, Hypophosphatämie

NCT04677439: rekrutiert derzeit Patienten für eine Phase-4-Studie in China: Wirksamkeit und Sicherheit von Flumatinib bei Patienten mit Ph+ CML-CP nach Imatinib-Versagen	Daten noch nicht verfügbar	Daten noch nicht verfügbar

*3G-TK1sVodobatinib:* neuartiges 3G-TKI mit begrenzter Off-Target-Aktivität, wirksam gegen natives und mutiertes *BCR::ABL* [107]		

NCT02629692: multinationale Phase-1/2-Studie bei mit Ponatinib behandelten und unbehandelten Patienten mit CML-CP, bei denen > 3 TKIs (oder weniger, falls andere zugelassene 3G-TKIs nicht infrage kamen) zur Bestimmung der MTD und RP2D versagten [96]	MTD (primärer Endpunkt): 204 mg; Wirksamkeit (sekundärer Endpunkt): MMR: 3/16 bei mit Ponatinib behandelten und 4/15 bei nicht mit Ponatinib behandelten Patienten, MCyR: 5/16 bei Ponatinib-behandelten Patienten, CCyR: 3/15 bei Ponatinib-naiven Patienten; Krankheitsprogression: 2/16 bei Ponatinib-behandelten und 4/15 bei Ponatinib-naiven Patienten	TEAEs Grad > 3 wurden bei > 1 mit Ponatinib behandelten Patienten berichtet, 2 (13%) jeweils Neutropenie, Amylase-Erhöhung und Thrombozytopenie; TEAEs Grad > 3, berichtet bei 7 (47%) der Ponatinib-naiven Patienten: je 1 Patient: Anämie, Lungenentzündung, Neutropenie, Gicht, Hypokaliämie und Thrombozytopenie, Demenz, Amnesie und erhöhte Leber- und Pankreasenzyme

*Olverembatinib:* ein neuartiges *BCR::ABL1*-TKI mit breitem Wirkungsspektrum, das gegen T3151-Mutationen aktiv ist [97]		

Phase-1-Dosiseskalations-/Erweiterungsstudie zur Bewertung der Sicherheit, vorläufigen Wirksamkeit und pharmakokinetischen und dynamischen Eigenschaften bei chinesischen Patienten mit TKI-resistenter CML-CP/AP [99]	CHR innerhalb von 3 Zyklen (primärer Endpunkt): CML-AP: 58% (n = 7/58); MCyR > 3 Zyklen (primärer Endpunkt): CML-CP: 54% (n = 21/58)	> 1 Grad 3–4 TRAE: 44 (63%) aller Patienten; dosisbegrenzende Toxizitäten: 2/3 der Patienten in der 60-mg-Kohorte

Phase-1-Dosiseskalations-/Expansionsstudie zur Bestimmung der MTD und der dosislimitierenden Toxizität bei chinesischen Patienten mit TKI-resistenter CML-CP/AP [98]	CHR: CML-CP: 95% (n = 52/55), CML-AP: 85% (n = 11/13); CCyR: CML-CP: 61% (n = 49/81), CML-AP: 36% (n = 5/14)	Die häufigsten Nebenwirkungen Grad > 3 bei > 10% der Patienten: Thrombozytopenie (n = 50/101, 50%), Leukopenie (n = 20/101, 20%), Anämie (n = 12/101, 12%)

*4G-TKI PF-114:* potenter 4G-TKI, selektiv gegen natives *BCR::ABL* und *BCR::ABL* mit T3151-Mutation [108]		

NCT02885766: Phase-1-Studie bei Patienten mit CML-CP/AP, bei denen > 2 TKIs versagten, oder mit *BCR:ABL1* TB151 mit > 6-monatiger Therapie zur Bestimmung der MTD und der dosislimitierenden Toxizität; Zwischenanalyse bei > 6 Monaten [101]	MTD (primärer Endpunkt): 600 mg; dosislimitierende Toxizität (primärer Endpunkt): 600 mg, die sich als Psoriasis-ähnliche Hautläsionen Grad 3 manifestieren; MCyR: 6/11 der Patienten, die eine Dosis von 300 mg erhielten, 4/12 der Patienten mit der *BCR:ABL1*-T3151-Mutation	Abbrüche aufgrund der Progression: n = 18/51 (35%); Abbruch aufgrund von UEs: n = 6/51 (12%); reversible Hauttoxizität Grad 3 (Psoriasis-ähnliche Hautläsionen): 11 Patienten > 400-mg-Dosis

*STAMP-InhibitorAsciminib:* neuartiger, erster STAMP-Inhibitor seiner Klasse, der an die Myristoyl-Tasche von *BCR:ABL* bindet [91]		

NCT03106779: Multizentrische Phase-3-Studie zum Vergleich von Asciminib und Bosutinib bei Patienten mit CML-CP, die zuvor mit > 2 TKIs behandelt wurden [109]	MMR nach 24 Wochen (primärer Endpunkt): Asciminib 26%, Bosutinib 13% (P = 0,029)	Grad > 3 TRAEs wurden bei 51% der mit Asciminib und 61% der mit Bosutinib behandelten Patienten berichtet; 1 Patient verstarb aufgrund behandlungsbedingter schwerwiegender UEs im Bosutinib-Arm

1G = erste Generation, 2G = zweite Generation, 3G = dritte Generation, 4G = vierte Generation, UE unerwünschtes Ereignis, AP = beschleunigte Phase, BID = zweimal täglich, CCyR = komplettes zytogenetisches Ansprechen, CHR = komplettes hämatologisches Ansprechen, CML-AP = chronische myeloische Leukämie in der akuten Phase, CML-CP = chronische myeloische Leukämie in der chronischen Phase, EMR = frühes molekulares Ansprechen, MCyR = gutes zytogenetisches Ansprechen, MMR = gutes molekulares Ansprechen, MTD = maximal tolerierte Dosis, ND = neu diagnostiziert, Ph+ = Philadelphia-Chromosom-positiv, STAMP = speziell auf die ABL-Myristoyl-Tasche abzielend, TEAE = bei der Behandlung auftretendes unerwünschtes Ereignis, TKI = Tyrosinkinase-Inhibitor, TRAE = behandlungsbedingtes unerwünschtes Ereignis.
